# Tetrahedral framework nucleic acids inhibit pathological neovascularization and vaso‐obliteration in ischaemic retinopathy via PI3K/AKT/mTOR signalling pathway

**DOI:** 10.1111/cpr.13407

**Published:** 2023-01-24

**Authors:** Xiaodi Zhou, Yanting Lai, Xiaoxiao Xu, Qiong Wang, Limei Sun, Limei Chen, Jiajie Li, Rong Li, Delun Luo, Yunfeng Lin, Xiaoyan Ding

**Affiliations:** ^1^ State Key Laboratory of Ophthalmology, Zhongshan Ophthalmic Center Sun Yat‐sen University, Guangdong Provincial Key Laboratory of Ophthalmology and Visual Science Guangzhou China; ^2^ Innovative Institute of Chinese Medicine and Pharmacy Chengdu University of Traditional Chinese Medicine Chengdu China; ^3^ State Key Laboratory of Oral Diseases, West China Hospital of Stomatology, Department of Maxillofacial Surgery, West China Stomatological Hospital Sichuan University Chengdu China

## Abstract

This study aimed to explore the effect and the molecular mechanism of tetrahedral framework nucleic acids (tFNAs), a novel self‐assembled nanomaterial with excellent biocompatibility and superior endocytosis ability, in inhibition of pathological retinal neovascularization (RNV) and more importantly, in amelioration of vaso‐obliteration (VO) in ischaemic retinopathy. tFNAs were synthesized from four single‐stranded DNAs (ssDNAs). Cell proliferation, wound healing and tube formation assays were performed to explore cellular angiogenic functions in vitro. The effects of tFNAs on reducing angiogenesis and inhibiting VO were explored by oxygen‐induced retinopathy (OIR) model in vivo. In vitro, tFNAs were capable to enter endothelial cells (ECs), inhibit cell proliferation, tube formation and migration under hypoxic conditions. In vivo, tFNAs successfully reduce RNV and inhibit VO in OIR model via the PI3K/AKT/mTOR/S6K pathway, while vascular endothelial growth factor fusion protein, Aflibercept, could reduce RNV but not inhibit VO. This study provides a theoretical basis for the further understanding of RNV and suggests that tFNAs might be a novel promising candidate for the treatment of blind‐causing RNV.

## Graphical abstract

The synthesis process of tetrahedral framework nucleic acids (tFNAs) and the mechanism of anti‐RNV by tFNAs. Four single‐stranded DNAs (ssDNAs) in equimolar amounts are successfully synthesized into tFNAs. tFNAs are capable to enter ECs, inhibit cell proliferation, tube formation and migration under hypoxic conditions and successfully reducing retinal neovascularization (RNV) and inhibit retinal vaso‐obliteration in oxygen‐induced retinopathy (OIR) model via regulating the PI3K/AKT/mTOR pathway.

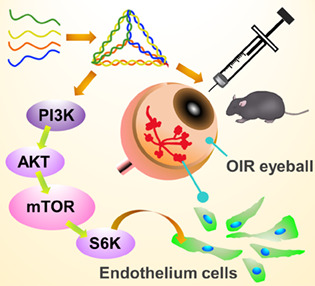

## 1 INTRODUCTION

Angiogenesis, the growth of new blood vessels from preexisting ones, plays a pivotal role in various physiological and pathological conditions.^1^ Pathological retinal neovascularization (RNV) is centrally involved in common and severe retinal diseases at all ages. For example, retinopathy of prematurity (ROP) is currently a major cause of acquired blindness in children,^2^, ^3^ proliferative diabetic retinopathy (PDR) accounts for the highest incidence of acquired blindness in the working‐age population,^4^, ^5^, ^6^ and retinal vein occlusion (RVO) represents one of the leading causes of blindness in people over the age of 65.^7^ The retina requires a continuous supply of oxygen (O_2_) and is known as one of the most metabolically active tissues, consuming O_2_ more rapidly than even the brain.^8^ Maintenance of an adequate oxygen supply is critical for retinal function. The high oxygen demands of the retina, and the relatively sparse nature of the retinal vasculature, are thought to contribute to the particular vulnerability of the retina to vascular disease. A large proportion of retinal blindness is associated with diseases having a vascular component, and disrupted oxygen supply to the retina is likely to be a critical factor.^9^

Currently, RNV patients with advanced ROP, PDR or RVO have been treated with retinal photocoagulation to decrease oxygen demand and relieve hypoxia,^10^, ^11^ or anti‐vascular endothelial growth factor (VEGF) therapy to inhibit neovascularization,^12^, ^13^, ^14^ while due to laser‐induced loss of retinal tissue, retinal photocoagulation can lead to complications, such as decreased visual acuity, diminished night vision and persistent visual field constriction.^15^ Anti‐VEGF therapy is the first‐line treatment for retinal neovascular diseases. It has been used clinically to treat hypoxia‐induced RNV in patients with ROP, DR, as well as RVO.^11^, ^16^, ^17^, ^18^, ^19^ However, anti‐VEGF drug has potential drawbacks. First, adverse effects associated with the blockade of VEGF signalling, including impairments of normal retinal vascular growth and retinal function, were suggested. Second, recurrence of RNV after intravitreal anti‐VEGF administration, due to the persistence of the ischaemic/non‐perfusion conditions,^11^, ^20^ is frequent in premature babies or diabetic patients.^11^, ^21^ Thus, there are highly desirable and, as‐yet, unmet medical needs, for alternative therapeutic strategies for treating RNV.

Recently, DNA nanotechnology has been developed and employed in a variety of fields owing to the extensive biological functions of DNA nanostructures.^22^, ^23^, ^24^, ^25^, ^26^ Tetrahedral framework nucleic acids (tFNAs) are synthesized by four isometric single‐stranded DNAs (ssDNAs) through a simple, rapid and reliable process^27^, ^28^, ^29^ which have been identified as three‐dimensional (3D) DNA nanostructures with a stable structure and superior mechanical properties.^30^, ^31^, ^32^, ^33^, ^34^ The tFNAs have been used extensively in various biological fields with considerable merits, including biocompatibility, structural stability and programmability.^35^, ^36^, ^37^, ^38^, ^39^, ^40^, ^41^, ^42^ For example, tFNAs could prevent apoptosis of neurons caused by oxygen–glucose deprivation/reoxygenation through interfering with ischemia in vitro and effectively ameliorate the microenvironment of the ischaemic hemisphere by upregulating expression of erythropoietin and inhibiting inflammation.^43^ We also found that tFNAs could promote angiogenesis in vitro and in vivo, via activation of Notch signalling, JAK/STAT signalling, as well as Akt/Nrf2/ HO‐1 Pathway.^44^, ^45^, ^46^, ^47^ Furthermore, tFNAs were found to be effective in ocular diseases prevention and treatment, for instance, for repairing corneal injury and suppress the oxidative stress in retinal ganglion cells.^48^, ^49^, ^50^ In addition, we previously showed that tFNAs, as vectors, inhibit choroidal neovascularization by polarizing macrophages.^51^ Hence, in this work, we chose tFNAs to explore the therapeutic effect and underlying mechanism in the treatment of RNV.

Therefore, we ventured to speculate that tFNAs can enter human umbilical vein endothelial cells (HUVECs) and regulate against neovascularization, for therapeutic purposes. Meanwhile, we also investigated the therapeutic effects of tFNAs on oxygen‐induced retinopathy (OIR) mouse models as well as the underlying mechanisms. Our results showed that tFNAs could effectively enter HUVECs and inhibit angiogenesis under hypoxic environment. More interestingly, we observed that tFNAs treatment on the ischaemic retina in OIR mouse models is beneficial not only for inhibiting pathological neovascularization, but also for ameliorating retinal avascular area and inhibiting retinal vaso‐obliteration (VO) area by modulating the PI3K/AKT/mTOR signalling pathway, suggesting that it may be a promising agent for the treatment of ischaemic RNV.

## 2 MATERIALS AND METHODS

### 2.1 Synthesis of tFNAs

Four ssDNA were synthesized and characterized by Genescript (Nanjing, China). First, TM buffer with a pH of 8.0 containing 10 mM Tris–HCl and 50 mM MgCl_2_ was prepared. Then, equal concentrations of the four ssDNAs were poured into TM buffer. After thorough mixing and centrifugation, the mixed solution was heated to 95°C for 10 min and then cooled to 4°C for 20 min to obtain tFNAs. The sequences of the four ssDNAs are shown in Table 1. Five hundred microlitres of tFNAs sample was concentrated to 100 μL with a 10 kD ultrafiltration tube and added to a high‐performance liquid chromatography (HPLC). Then, tFNAs were purified with a DNA Pac™PA100 (Thermo Scientific, USA) chromatographic column at a flow rate of 1 mL min^−1^ with different mobile phases (mobile phase A:25 mM Tris–HCL, mobile phase B: 25 mM Tris–HCL + 375 mM NaClO_4_). Next, the main peak of the sample was collected, and polyacrylamide gel electrophoresis (PAGE) was used for purity detection.

**TABLE 1:** The sequences of the four single‐stranded DNAs (ssDNAs)

ssDNA	Base sequence (5′–3′)
S1	ATTTATCACCCGCCATAGTAGACGTATCACCAGGCAGTTGAGACGAACATTCCTAAGTCTGAA
S2‐Cy5	ACATGCGAGGGTCCAATACCGACGATTACAGCTTGCTACACGATTCAGACTTAGGAATGTTCG
S3	ACTACTATGGCGGGTGATAAAACGTGTAGCAAGCTGTAATCGACGGGAAGAGCATGCCCATCC
S4	ACGGTATTGGACCCTCGCATGACTCAACTGCCTGGTGATACGAGGATGGGCATGCTCTTCCCG

### 2.2 Characterization of tFNAs

We conducted experiments using the methods described in previous studies to verify the successful synthesis of tFNAs. Briefly, we used PAGE and high‐performance capillary electrophoresis (HPCE) to detect the difference in the molecular weights of tFNAs and then used a nanoparticle size analyser to detect the difference between the zeta potential and particle sizes of the two reagents to determine whether the synthesis was successful.

### 2.3 Cell culture and treatment

HUVECs were purchased from ATCC. We incubated the cells with 1 × Dulbecco's Modified Eagle's Medium Nutrient Mixture F‐12(DMEM/F‐12 basic [Gibco, USA]) supplemented with 10% fetal bovine serum [FBS (Gibco, USA)], and 100% antibiotic solution (10,000 U mL^−1^ penicillin and 10,000 μg mL^−1^ streptomycin) (Gibco, USA) in normoxia environment comprising 95% air and 5% CO_2_ at 37°C for 24 h. Next, HUVECs were cultured under normoxia (37°C, 5%CO_2_) or hypoxia (37°C, 1% O_2_, 5%CO_2_) with various concentrations (vehicle, 100 nmol L^−1^ of tFNAs or 1 μg μL^−1^ of Aflibercept [AFL]), and the original DMEM/F‐12 containing 10% FBS accordingly for another 24 h.

### 2.4 Uptake of Cy5‐loaded‐tFNAs

In this experiment, we tested whether tFNAs successfully entered HUVEC cells. We modified one of the ssDNAs (S2) labelled with Cy5 fluorescence in the tFNAs. HUVEC cells were cultured on a 6‐well plate at the density of 4 × 10^5^ cells per well. After the cells were cultured for 24 h, the Cy5‐tFNAs were added and cultured for 8–24 h, respectively. We then measured the fluorescence intensity at 8 h and compared it with the blank control group. And samples meeting the requirements of flow cytometry were collected. Flow cytometry (Attune NxT, ThermoFisher Scientific, USA) was used to spot the intracellular fluorescence intensity at 24 h.

### 2.5 Cell immunofluorescence assay

Cell immunofluorescence staining was conducted to evaluate the expression of proteins related to angiogenesis and cell apoptosis. HUVECs were plated in 6‐well plates (Corning, USA) at a density of 9.6 × 10^5^ cells and treated as mentioned above. Then, the cells were fixed with 4% paraformaldehyde (PFA) in PBS for 15 min, permeabilized and blocked with 0.5% Triton X‐100 (Biofroxx, Germany) and 1% BSA (Biosharp, Anhui, China) for 20 min. Then, the samples were incubated together with the following diluted vascular endothelial growth factor 2 (VEGFR2) (1:200, YT5845, Immunoway) and S6K (1:200, YT3555, Immunoway) primary antibody at 4°C overnight. Next, they were cultured with secondary antibody anti‐rabbit IgG [1:1000, 4413, Cell Signalling TECHNOLOGY(CST)] under ambient temperature for 2 h. Then, the cell nucleus was stained with Hoechst 33342 (Solarbio, Beijing, China), and the cytoskeleton was stained with phalloidin (Solarbio, Beijing, China). Samples were fixed with anti‐fluorescence quenching sealed tablets.

### 2.6 Real‐time fluorescence quantitative PCR

We used real‐time fluorescence quantitative PCR (RT‐PCR) technology to detect the expression of related genes. The total RNA was extracted with TRNzol Universal (TIANGEN, Beijing, China). Then, used the FastKing RT Kit With gDNase (TIANGEN, Beijing, China) to purify and reverse transcription. All target mRNAs were amplified by quantitative RT‐PCR using SYBR Green Realtime PCR Master Mix (TOYOBO, Shanghai, China). Table 2 lists the corresponding primers for the genes (VEGF, HIF‐1α, PI3K, AKT and mTOR), all of which are BLAST search design with β‐actin amplification as the control.

**TABLE 2:** The sequences of PCR primers

Gene	Primer sequence (5′‐3′)
VEGF	Forward: GAGCAACATCACCATGCAGATCA Reverse: AACCGGGATTTCTTGCGCT
PI3K	Forward: CAGCACTGCCTCCTAAACCA Reverse: GTCCCGTCTGCTGTATCTCG
AKT	Forward: TGCACAAACGAGGGGAATATAT Reverse: CGTTCCTTGTAGCCAATAAAGG
mTOR	Forward: TGGTGCGACACCGAATCAAT Reverse: TTGGCCACTCCTAAGCATCC
HIF‐1α	Forward: GAACAAAACACACAGCGAAGC Reverse: TGTGCAGTGCAATACCTTCCA

### 2.7 Protein extraction and western blot analysis

HUVECs were cultured, divided into groups, and treated accordingly. After 24 h, protein was extracted from the HUVECs with RIPA (Solarbio, Beijing, China) containing protease inhibitors. Protein concentrations were determined by the BCA Protein Quantitation Assay Kit (KeyGEN, Nanjing, China) using protein standard solution as a standard. Samples of supernatants containing 20 μg protein were heated to 95°C for 5 min and separated with SurePAGE™, Bis‐Tris, 10 × 8, 4%–20%, 12 wells (GenScript, USA) and transferred to polyvinylidene difluoride filter (PVDF) membrane (Millipore, Merck, Germany). After blocking with 5% defatted milk in TBS‐Tween‐20 (TBST) for 1 h at room temperature (RT), the PVDF membranes were incubated with diluted VEGFR2 (1:1000, YT5845, Immunoway), mTOR (1:1000, 2983 S, CST), p‐mTOR (1:1000, 5536 S, CST), HIF‐1α (1:1000, 36169 S, CST), PI3K (1:1000, 4257 S, CST), p‐PI3K (1:1000, 4228 S, CST), AKT (1:1000, ET1609, huabio), p‐AKT (1:1000, ET1607, huabio), S6K (1:1000, A16968, Abclonal), p‐S6K (1:1000, AP1106, Abclonal) and β‐actin (1:1000, 4967 S, CST) antibodies in blocking solution overnight at 4°C. On the following day, after washing for five times, the PVDF membranes were then incubated with the anti‐rabbit IgG, Horseradish peroxidase (HRP)‐linked antibody (1: 3000, 7074, CST) at RT for 2 h, and visualized using an enhanced chemiluminescence system (ProteinSimple, USA).

### 2.8 Angiogenesis experiments

#### 2.8.1 EdU cell proliferation assay

To confirm that cell proliferation was inhibited by tFNAs, 5‐Ethynyl‐2'‐deoxyuridine (EdU) was detected using Alexa Fluor 488 Click‐iT EdU Imaging Kits (ThermoFisher Scientific, USA). HUVECs were seeded in 6‐well plates and cultured for 24 h, following by treatments described in ‘Cell culture and treatment’. EdU cell proliferation assay was performed according to the manufacturer's instructions. The fluo in 6‐well plates staining images of HUVECs were observed by a confocal laser microscope (Carl Zeiss, Oberkochen, Germany). ImageJ software was used for statistical analysis. Experiments were performed a minimum of three times.

#### 2.8.2 Tube formation assay

Tube formation experiment was performed to explore the influence of tFNAs on HUVECs angiogenesis. HUVECs were seeded in 6‐well plates and cultured for 24 h. Matrigel solution (50 μL per well) was added to a 96‐well plate (kept on ice) and incubated at 37°C for 30 min to allow gel formation. Fifty microlitres of HUVECs (approximately 1.2 × 10^5^ cells mL^−1^) with vehicle, 45 μL of HUVECs (approximately 1.2 × 10^5^ cells mL^−1^) with 5 μl of 1uM of tFNAs (100 nmol·L^−1^) and 48.75 μL of HUVECs (approximately 1.2 × 10^5^ cells mL^−1^) with 1.25 μL of 40 mg mL^−1^ of AFL (1 μg μL^−1^) were seeded in the prepared 96‐well plates (containing gels). After incubation under normoxia (37°C, 5%CO_2_) or hypoxia (37°C, 1% O_2_, 5%CO_2_) with the original DMEM/F‐12 containing 10% FBS accordingly. Twelve hours later, six different wells per group were photographed using the Inverted Biologic Microscope (IX2‐SL, Olympus Corporation, Japan). Then, tube formation on images was quantified using the Angiogenesis Analyser plugin for ImageJ. Experiments were performed a minimum of three times.

#### 2.8.3 Cell migration assay

To explore the influence of tFNAs on HUVECs migration, we conducted a wound healing experiment. HUVECs were seeded in 6‐well plates and cultured for 24 h. The cells were divided into 6 groups and appropriate treatments (vehicle, 100 nmol L^−1^ of tFNAs, or 10 mg mL^−1^ of AFL) were added to the culture medium (without growth factors or FBS). Then, a scratch was made with a sterile pipette tip, and wound images were taken after incubation under normoxia (37°C, 5%CO_2_) or hypoxia (37°C, 1% O_2_, 5%CO_2_) for 0, 24 and 48 h under the Inverted Biologic Microscope (IX2‐SL, Olympus Corporation, JAPAN). ImageJ software was used for statistical analysis. Experiments were performed a minimum of three times.

### 2.9 OIR model

OIR is the most widely studied model of a retinal disease featuring pathological RNV and is a well‐established model for investigating novel putative antiangiogenic compounds. C57BL/6J mice were purchased from GemPharmatech Co., Ltd (Nanjing, China). All animal studies were approved by the Institutional Animal Care and Use Committee of Zhongshan Ophthalmic Center. OIR was carried out in C57BL/6J mice as previously described by Smith et al.^52^ and our previous studies.^53^ This model involves placing nursing postnatal day 7 (P7) mice with their mothers in a 75% O_2_ chamber until P12, which suppresses the still‐ongoing normal retinal vascular development by destroying many endothelial and astrocytic cells, so that several retinal areas become VO. When the mice are returned to room air (21% O_2_), the relative hypoxia over the subsequent 5–7 days promotes excess VEGF production and leads to a pathological increase in retinal angiogenesis. Tufts of new blood vessels characteristic of the pathological RNV form on the inner surface of the retina.

### 2.10 Intravitreal injection

First, the mice were anaesthetised with 1% pentobarbital sodium (50 mg kg^−1^). Next, 1 μL of tFNAs (1 μmol L^−1^) or AFL (10 μg μL^−1^) was injected into the vitreous with a 33‐gauge Hamilton syringe (Hamilton, USA) under a stereomicroscope (M620 F20, Leica Microsystems, France) to avoid lens injury at P12 in OIR mice or normoxia animals. The control group was intravitreally injected 1 μL of vehicle. Then, tobramycin eye ointment was applied to prevent infection after the operation.

### 2.11 Whole‐mounted retinal immunofluorescence

Mice were euthanized at P17, and eyes were enucleated and fixed with freshly prepared 4% PFA for 1 h. After being dissected under a stereo operating microscope (MZ62, Mshot, Guangdong, China), intact retinas were blocked and permeabilized in PBS containing 1% BSA and 0.5% Triton X‐100 and were incubated with primary antibodies for IB4 conjugated to AlexaFluor 488 (1:200, I21411, ThermoFisher), overnight at 4°C. Retinas were then washed with PBS, mounted on slides and then analysed by inverted microscope (Ts2FL, Nikon, Japan).

### 2.12 Measurement of RNV

To determine the extent of RNV, retinal images from tFNAs or AFL‐treated and vehicle‐injected eyes were randomized, labelled, and analysed for RNV clock hours and percent RNV area to total retinal area. Two masked reviewers performed all analyses. The presence of RNV was determined with a technique adapted from those used in clinical trials^54^ and animal model determination.^55^ For clock hours, flat mounts were divided into 12 clock hours of approximately equal area using Adobe Photoshop, assessed for the presence of RNV.^54^, ^55^ RNV area and total retinal area were quantified using ImageJ. The area in each clock hour exhibiting RNV was measured and added together as the total RNV area per retina and expressed as a percent of the total area of the retina.

### 2.13 Retinal frozen sectional immunofluorescence

To make retinal frozen sections, eyes of mice were enucleated and fixed in 4% PFA for 1 h at RT. Next, eyes were dehydrated in 10%, 20% and 30% sucrose (Biofroxx, Germany) in PBS accordingly and embedded in O.C.T. (SAKURA, Japan) compound. Then they were fast frozen and cut into 6 μm‐thick sections. The sections were washed with PBS, and then blocked and permeabilized with 1% BSA and 0.5% Triton X‐100 for 2 h at RT. The sections were incubated with IB4 conjugated to AlexaFluor 488 (1:200, I21411, ThermoFisher) or primary antibodies for CD31(1:100, sc‐376764, Santa Cruz) overnight at 4°C. After washing in PBS, sections were incubated for 2 h at RT with secondary antibody anti‐rabbit IgG (1:1000, 4413, CST) and were counterstained with Hoechst 33342 (Solarbio, Beijing, China) for 10 min. The frozen retinal sections were then analysed by confocal microscopy (Carl Zeiss, Oberkochen, Germany).

### 2.14 Statistical analysis

Statistical analyses were performed using SPSS Statistics 25.0. Data from multiple repeat experiments are presented as the means ± SD. Significant differences among the groups were evaluated by one‐way analysis of variance (ANOVA), and *p* < 0.05 was considered statistically significant. The number of replicates and/or the total number of animals are shown in the figure legends or within the figures.

## 3 RESULTS

### 3.1 Synthesis and characterization of tFNAs

**FIGURE 1:**
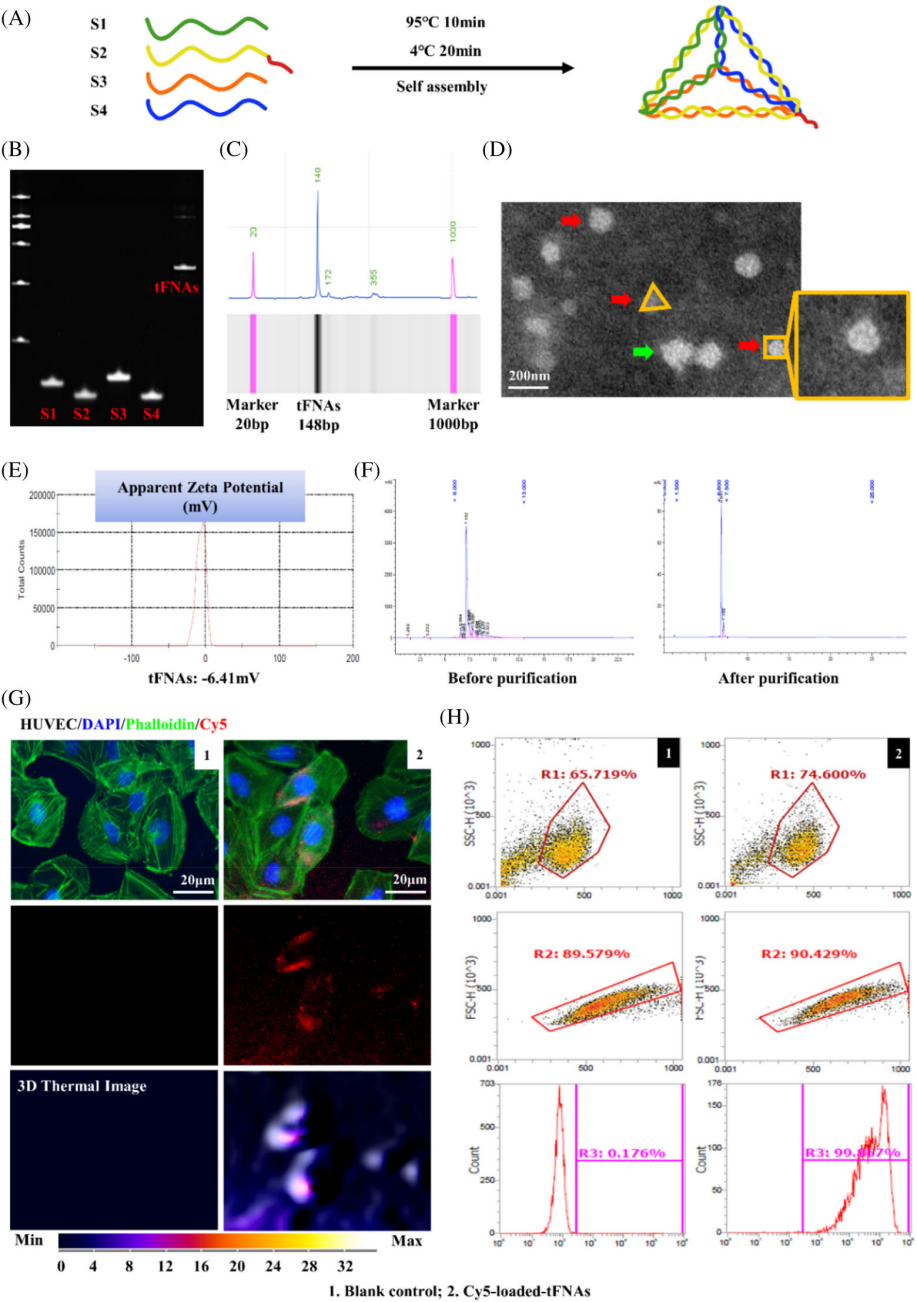
Synthesis and characterization of tetrahedral framework nucleic acids (tFNAs). (A) The synthesis of tFNAs. (B) Molecular weights of the synthesized tFNAs detected by polyacrylamide gel electrophoresis (PAGE). (C) Molecular weight of the synthesized tFNAs detected by high‐performance capillary electrophoresis (HPCE). (D) Transmission electron microscope (TEM) image of molecular structure of the synthesized tFNAs. The red arrows point to successfully synthesized tFNAs. The green arrows point to polymers. Scale bars are 200 nm. (E) Stability of tFNAs measured by zeta potential analysis. (F) The result of high‐performance liquid chromatography (HPLC) of tFNAs before and after purification to remove mismatched bases or single strand. (G) The uptake of tFNAs by human umbilical vein endothelial cells (HUVECs) after 8 h. Red: Cy5‐loaded‐tFNAs; Blue: nuclei; Green: cytoskeleton. Scale bars are 20 μm. (H) Flow cytometry to see the uptake of tFNAs by HUVECs. The rate of endocytosis was 0.176% in blank control group with vehicle and 99.867% in group with tFNAs at 24 h.

Four ssDNAs in equimolar amounts shown in Table 1 were successfully synthesized into tFNAs. Each ssDNA was further divided into three small fragments self‐assembled to form one triangle facet and hybridized with the other three strands according to highly specific complementary base‐pairing. A brief description of the synthesis of tFNAs is provided (Figure 1A). Among the ssDNAs, the fluorescent molecule Cy5 designed on the S2 chain was used to show the localization of tFNAs in subsequent experiments. The results of 8% polyacrylamide gel electrophoresis (PAGE) verified that we had succeeded in synthetizing tFNAs. According to the theoretical value in previous studies that tFNAs are comprised of four ssDNA molecules (Figure 1B).^56^ In addition, high‐performance capillary electrophoresis (HPLC) was used to detect the successful synthesis of tFNAs (Figure 1C), and the results were consistent with those obtained by PAGE.

Next, we examined the characterization of tFNAs by transmission electron microscopy (TEM) (Figure 1D). The result indicated that, similar to the results in previous studies,^35^, ^49^ the successfully synthesized tFNAs were triangle like. The particle size of tFNAs was approximately 19.30 nm. As nucleic acids have a negative charge, their zeta potential was approach to −6.41 mV (Figure 1E), confirming the stability of the synthesized tFNAs. The results showed that the purity of tFNAs was effectively improved (Figure 1F).

### 3.2 Cellular uptake of tFNAs

We validated the ability of tFNAs to enter HUVECs. HUVECs were treated with Cy5‐loaded‐tFNAs for 8 h and determined the localization of tFNAs via immunofluorescence assay. It could be observed that tFNAs were already present in the cytoplasm (Figure 1G). Cells were then collected for flow cytometry analysis after 24 h Cy5‐loaded‐tFNAs treatment, and the results showed that the entry rate of tFNAs into cells was 99.867% while the entry rate in control group was only 0.176% (*p* < 0.001, Figure 1H).

### 3.3 tFNAs inhibit HUVECs cell proliferation, tube formation and migration in vitro

**FIGURE 2:**
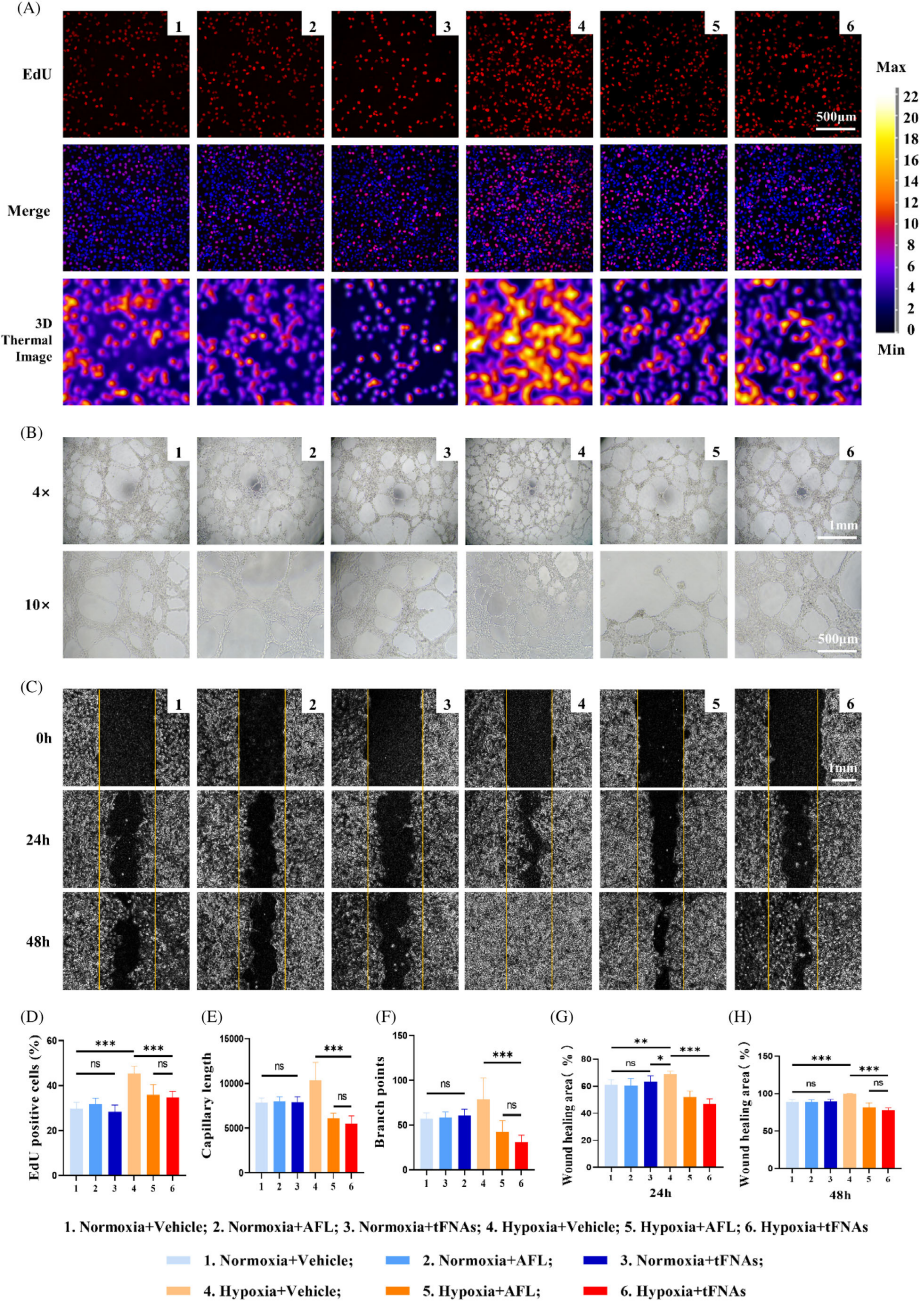
Tetrahedral framework nucleic acids (tFNAs) inhibit angiogenesis in vitro. (A) The results of cell proliferation assay of human umbilical vein endothelial cells (HUVECs) after different treatments. Scale bars are 500 μm. (B) The results of tube formations of HUVECs after different treatments. Scale bars are 500 μm and 1 mm. (C) The binary Image results of scratch‐wound assay using HUVECs with at 0, 24 and 48 h after different treatments. Scale bars are 500 μm. (D) Analysis of quantification of percentage of Hoechst (blue) positive nuclei colocalized with EdU (red). Data are presented as mean ± SD (*n* = 6). (E) Analysis of the capillary lengths. Data are presented as mean ± SD (*n* = 6). (F) Analysis of branch points. Data are presented as mean ± SD (*n* = 6). (G) Analysis of the wound healing area rates at 24 h. Data are presented as mean ± SD (*n* = 6). (H) Analysis of the wound healing area rates at 48 h. Data are presented as mean ± SD (*n* = 6). Statistical analysis: the ANOVA test was applied, ***p ≤ 0.001, **p ≤ 0.01 and *p<0.05.

First, HUVECs were then cultured with vehicle or 1 μg μL^−1^ Aflibercept (AFL) or 100 nmol L^−1^ tFNAs under normoxia or hypoxia (37°C, 1% O_2_, 5%CO_2_) for 24 h. The EdU assay was used to examine cell proliferation (Figure 2A). The results showed, no significant difference among 3 normoxic groups (Figure 2D). However, compared to the vehicle group (45.44% ± 3.03%), the proliferation of HUVECs in AFL (35.81% ± 4.68%) and tFNAs (34.66% ± 2.64%) were significantly less active. These results indicated that hypoxia‐induced cell proliferation could be ameliorated by tFNA treatments.

Then, we cultured HUVECs 12 h for investigating tube formation under normoxia or hypoxia conditions (Figure 2B). No significant differences were found in capillary length nor in branch points among vehicle group (7831.33 ± 535.09 and 57.00 ± 6.57), AFL group (7995.67 ± 518.84 and 60.67 ± 6.89) and tFNAs group (7892.00 ± 625.46 and 58.00 ± 6.45) (Figure 2E, F). While under hypoxic condition, the results showed that the capillary length (10372.83 ± 1953.03) and branch points (78.67 ± 24.04) of HUVECs increased microscopically (Figure 2E, F). More importantly, the number of them could be significantly reduced after AFL treatment (6078.00 ± 597.98 and 42.50 ± 6.57) or tFNAs treatment (5507.33 ± 877.73 and 57.00 ± 6.57), and there was no significant difference between AFL and tFNAs groups (*p* = 0.326 and *p* = 0.114, Figure 2B, E, F). These suggested that tube formation of HUVECs could be effectively inhibited by tFNAs.

Finally, scratch‐wound assay was used to further detect the migration ability of HUVECs at 24 and 48 h. Under normoxic conditions, no significant migration was observed in AFL or tFNAs‐treated HUVECs compared with that in the vehicle group (all *p* > 0.5, Figure 2C, G, H). However, quantitative analysis of wound healing area rates showed less migration in tFNAs (47.00% ± 3.79% and 78.17% ± 2.64%) when compared with AFL‐treated cells (52.00% ± 4.34% and 81.67% ± 6.15%), while the rates in vehicle group were (68.83% ± 2.14% and 99.56% ± 0.40%) of hypoxic HUVECs after different treatments at 24 h and 48 h respectively could be observed (Figure 2C, G, H).

### 3.4 tFNAs reduce angiogenesis and inhibit the VO in OIR model

**FIGURE 3:**
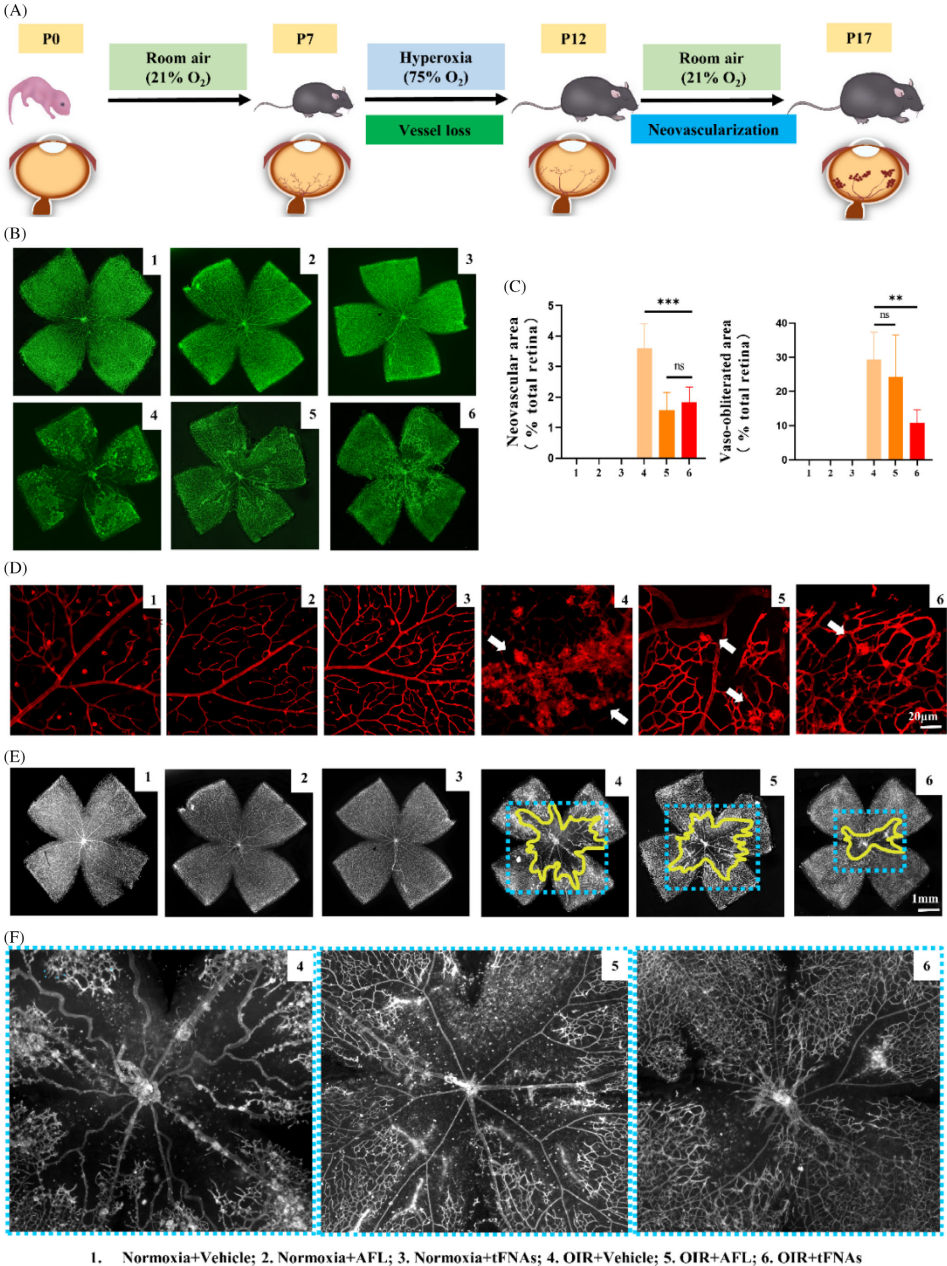
tFNAs reduce avascular area and inhibit pathological angiogenesis in OIR model on retinal flat‐mount. **(**A) Description of the OIR model in vivo experiment of retinal anatomy. (B) The results of IB4 staining. Scale bars are 1 mm. NV: neovascularization, AFL, aflibercept, OIR, oxygen‐induced retinopathy; tFNAs, tetrahedral framework nucleic acids. (C) Analysis of neovascular area and avascular area. Data are presented as mean ± SD (*n* = 7–8). (D) The neovascular sprouts (white arrow) in different groups after different treatments. Scale bars are 20 μm. (E) The vaso‐obliterated area (VO) portrayed by yellow line in OIR model. (F) The magnified VO area (blue dot line). Scale bars are 1 mm. Statistical analysis: the ANOVA test was applied, ***p ≤ 0.001, **p ≤ 0.01 and *p< 0.05.

The OIR model was carried out in C57BL/6J mice. Then, intravitreous vehicle (1 μL), AFL (10 μg μL^−1^) or tFNAs (1 μmol L^−1^) were injected at postnatal 12 (P12) to investigate the potential therapeutic relevance of tFNAs (Figure 3A). Mice under normoxia, served as controls, were received the same treatment. The results indicated that under normoxia, the retinal vessels have no difference before and after the treatments (Figure 3B–D). Compared with vehicle‐treated group (3.60% ± 0.79%), both AFL (1.58% ± 0.57%) and tFNAs (1.83% ± 0.50%) could effectively reduce the percentage of RNV area in OIR mice (*p* < 0.001) and there was no significant difference between these two groups (*p =* 0.433, Figure 3B–D).

**FIGURE 4:**
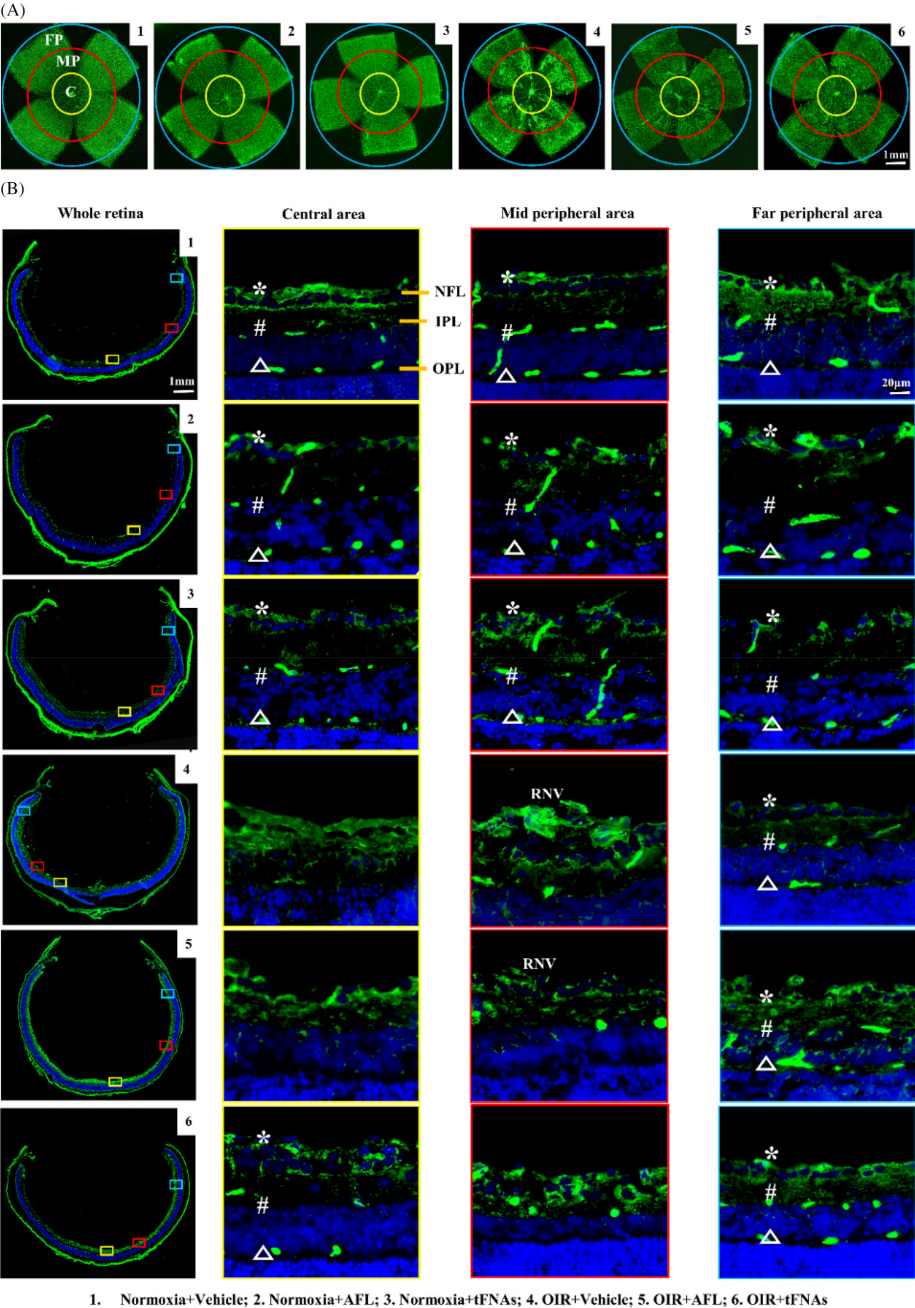
tFNAs reduce avascular area and inhibit pathological angiogenesis in OIR model on retinal cross‐section. (A) Different zones on immunofluorescent staining retinal flat mounts. Green: retinal mounts stained with IB4. (C) Central area, FP, far peripheral area; MP, mid peripheral area. Scale bars are 1 mm. (B) Different zones on immunofluorescent staining retinal cross‐sections. Green: vessels stained with CD31, Blue: cell nuclei with Hoechst. SP (*): superficial plexus, IP (#): intermediate plexus, DP (△): deep plexus, ILM, inner limiting membrane; IPL, inner plexiform layer; NFL, nerve fibre layer; OPL, outer plexiform layer. Scale bars are 1 mm and 20 μm. All the three plexuses were unremarkable in three normoxia groups in C, MP and FP areas. In nomoxia mice, SP were located in NFL, IP in IPL and DP in OPL (Columns 1–3). In vehicle‐treated OIR mice, no plexus was found in C. RNV sprouted breakthrough ILM. IP and DP were absent in FP (Column 4). In AFL‐treated OIR mice, absence of three plexuses were noted in C. However, in tFNAs, vessels were found regenerated with relative normal structures (Columns 5 and 6).

The VO area was significantly decreased after tFNAs treatment (10.90% ± 3.73%, *p* = 0.001), while no decrease in AFL group (24.23% ± 12.26%, *p* = 0.715), when compared with that in vehicle‐treated group (29.44% ± 7.89%) (Figure 3B, C, E, F). We then investigated the presence and location of the vessels regenerated in VO area. The retina whole‐mount was divided into three areas, central areas (C), mid‐peripheral (MP) area and far peripheral (FP) area (Figure 4A). With cross‐section, three layers of retinal vascular plexus can be identified. Briefly, the superficial plexus (SP) were located in nerve fibre layer (NFL), the intermediate plexus (IP) in the inner plexiform layer (IPL) and the deep plexus (DP) in the outer plexiform layer (OPL).^57^ In this study, all the three plexuses were unremarkable in three normoxia groups, even after AFL or tFNAs treatment (Figure 4B). Moreover, RNV sprouted breakthrough the inner limiting membrane (ILM) in the vehicle‐treated OIR group. All three layers of vascular structures disappeared in central area (Figure 4B). In AFL‐treated OIR mice, no vessels were noted in central area. Significant decrease of RNV was found in MP area with disappearance of IP and DP. In tFNAs‐treated OIR mice, regenerated SP could be seen, with some IP and DP. In the MP area, fewer RNV was noted if compared with AFL in the MP area. In addition, there were no significant difference in the FP area among all groups (Figure 4B).

### 3.5 tFNAs ameliorate cell proliferation, migration and reduce RNV via the PI3K/AKT/mTOR pathway

**FIGURE 5:**
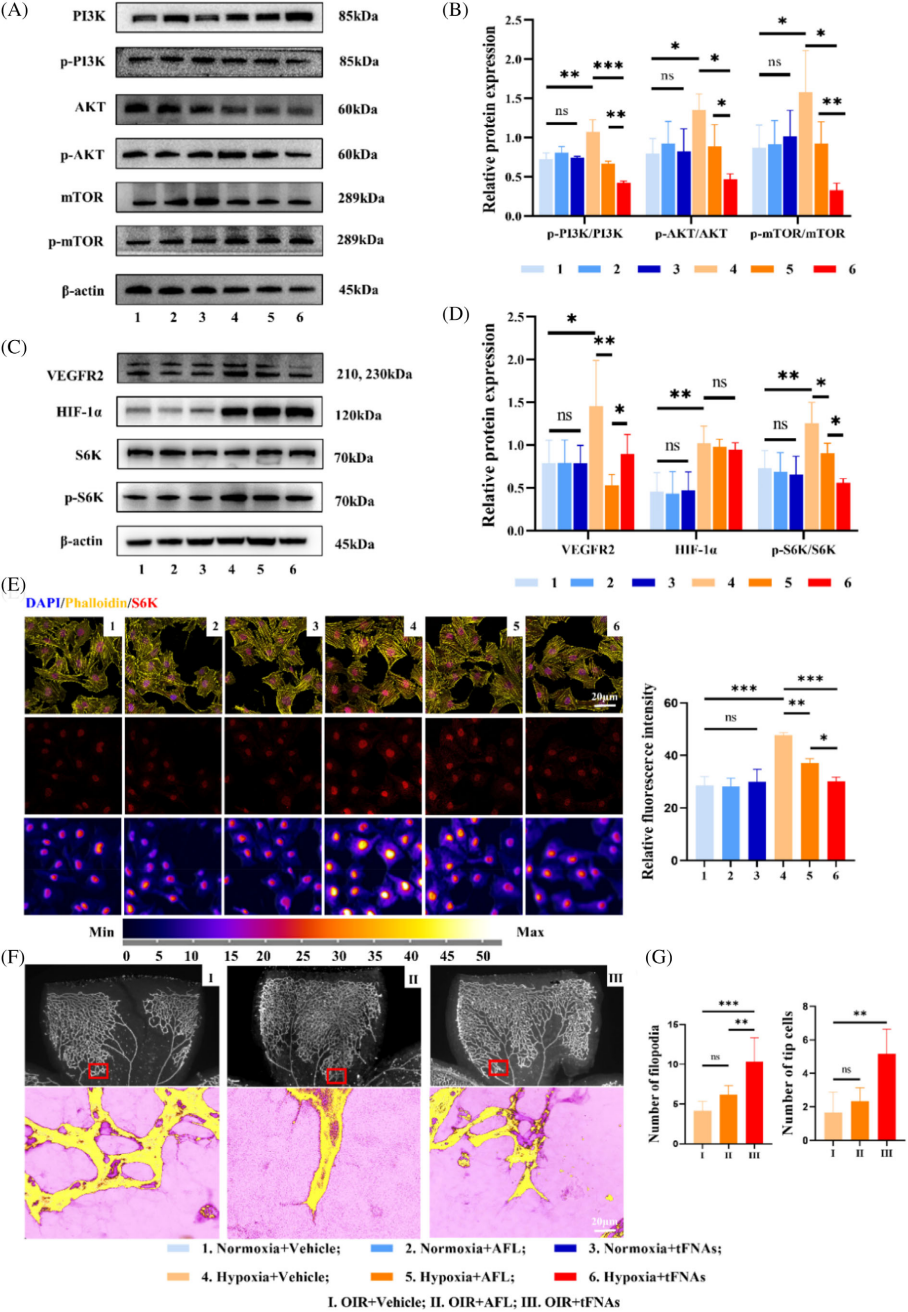
tFNAs prevent HUVECs proliferation and reduce avascular area via the PI3K/AKT/mTOR pathway. **(**A) Western blot analysis of the PI3K, p‐PI3K, AKT, p‐AKT, mTOR and p‐mTOR expression levels (β‐actin was used as an internal control). (B) The relative protein expression intensity of p‐PI3K/PI3K, p‐AKT/AKT, p‐mTOR/mTOR. Data are presented as mean ± SD (*n* = 3). (C) Western blot analysis of the VEGFR2, HIF‐1α, S6K and p‐S6K expression levels (β‐actin was used as an internal control). (D) The relative protein expression intensity of VEGFR2, HIF‐1α and p‐S6K/S6K. Data are presented as mean ± SD (*n* = 3). (E) The expression of S6K (red) and its co‐location with phalloidin (yellow) were detected by immunofluorescence. Statistics of relative fluorescence intensity of S6K. All data are presented as mean ± SD (*n* = 3). Scale bars are 20 μm. (F) IB4 staining of OIR + vehicle, OIR + AFL and OIR + TDN retinas processed by ZEN lite 3.3 at D14 after injections. Yellow: IB4 stained vessels, Purple: filopodia. Scale bars are 20 μm. (G) Analysis of number of filopodia and tip cells. Data are presented as mean ± SD (*n* = 6). Statistical analysis: The ANOVA test was applied, ***p ≤ 0.001, **p ≤ 0.01 and *p<0.05.

To confirm whether tFNAs ameliorate cell proliferation and migration via the PI3K/AKT/mTOR/S6K pathway in this study, the expression levels of VEGFR2, AKT, PI3K subunits, mTOR and S6K in normoxic or hypoxic HUVECs were detected by western blot analysis (Figure 5A, B). In our results, HIF‐1α, VEGFR2, p‐PI3K/PI3K, p‐AKT/AKT, p‐mTOR/mTOR and p‐S6K/S6K increased conspicuously under hypoxia (Figure 5A–D), and dramatically decreased after AFL and tFNAs treatment (all *p* < 0.05). Meanwhile, increased S6K immunofluorescence (red, Figure 5E) was detected in vehicle‐treated hypoxic HUVECs, but significant decreased in AFL or tFNAs‐treated cells (Figure 5E). However, p‐PI3K/PI3K, p‐AKT/AKT, p‐mTOR/mTOR and p‐S6K/S6K deceased significantly more in tFNAs group, when compared with those in AFL group (*p* < 0.05 in p‐AKT/AKT and p‐S6K/S6K*, p* < 0.01 in p‐PI3K/PI3K and p‐mTOR/mTOR) (Figure 5A–D), while VEGFR2 was inhibited more in AFL group, when compared with that in tFNAs group (*p<* 0.05, Figure 5C,D). Further confocal micrograph showed the extensions of filopodia at the leading edge of growing blood vessels at the edge of VO and we found that the number of filopodia (10.33 ± 3.01) and tip cells (5.17 ± 1.47) were significantly increased in tFNAs‐treated cells, but not in AFL‐treated cells (Figure 5F, G).

## 4 DISCUSSION

RNV is one of the leading irreversible causes of serious visual impairment and blindness in millions of patients worldwide. It is the cause of ROP, PDR and RVO.^2^, ^3^, ^4^, ^5^, ^6^, ^7^ Current mainstream therapies such as anti‐VEGF drugs and photo coagulation has their potential drawbacks due to complications caused by blockade of VEGF signalling, persistence of the ischaemic/non‐perfusion condition and loss of retinal tissue.^11^, ^12^, ^13^, ^14^, ^15^, ^20^

In our study, we explored the treatment effect and the molecular mechanism of tFNAs in ischaemic retinopathy. In consistent with previous studies, DNA nanomaterials also exhibit excellent stability and biological applications among similar materials.^35^, ^49^, ^56^ To better demonstrate the effect of tFNAs and exclude confounding factors, we purified the synthesized tFNAs before experiments and tested their purity using HPLC before and after purification. After purification, supernumerary and mismatched bases and single strands were removed, which may be responsible for the discrepancy in the best concentration between in vivo experiments in this study and those (250 nmol L^−1^) in previous studies.^51^

We confirmed in different ways that tFNAs were successfully synthesized and taken up by cells, which was the foundation for our subsequent experiments. As previously reported, DNA nanostructures have many excellent characteristics, which make them widely used in biological and biomedical applications.^58^, ^59^ The most outstanding characteristic of tFNAs compared to that of ssDNA and other spatial nanostructures, is that they enhanced endocytosis remarkably.^42^ Importantly, tFNAs were noted to penetrate the cellular membrane without the help of transfection agents. Moreover, Fan et al. first used the single‐particle tracking technique to report that Cy3‐labelled tFNAs adjust their orientation so that their corners attach to the cell membrane, minimizing charge repulsion and leading to charge redistribution; this attachment is followed by the endocytosis of tFNAs via the caveolin‐mediated pathway and their entry into lysosomes in a microtubule‐dependent manner.^24^, ^42^, ^60^ In our study, dose‐effect experiment was conducted, and VEGF was most inhibited at the concentration of 100 nmol L^−1^ (purified), instead of unpurified 250 nmol·L^−^1.^44^, ^45^, ^46^, ^47^ Thus, purified 100 nmol L^−1^ tFNAs were employed in all in vitro and in vivo experiments. Several proangiogenic factors are consistently upregulated during diverse forms of pathological angiogenesis, including two members of the VEGF family, VEGF‐A and placental growth factor (PlGF).^61^ Hypoxia induced an increase of HIF‐1α, and also increased expression of vascular endothelial growth factor A (VEGFA) and VEGFR‐2 protein through the HIF‐1α/VEGFA/VEGFR‐2 axis.^62^, ^63^ These factors activate quiescent endothelial cells and promote cell proliferation, migration and vascular permeability. Previous studies indicated that AFL could inhibit these pathological cell behaviours as a decoy receptor for all diffusible isoforms of VEGF‐A, VEGF‐B and PIGF.^61^, ^64^, ^65^, ^66^ As the main function of HUVECs, angiogenesis refers to the complex process by which new blood vessels are constructed and involves cell proliferation, tube formation and migration in hypoxia associated RNV and is shown as VEGF dependent. Several cellular functional assays, including EdU cell proliferation assays, tube formation assays and wound healing assays were conducted, and the results showed that tFNAs significantly ameliorated proliferation, migration and tube formation of HUVECs in vitro.

The OIR mouse model of hypoxia‐induced NV is a well‐established model for investigating novel putative antiangiogenic compounds.^52^, ^67^ In the in vivo animal experiment, a mouse model was conducted as previously described by our study. In this study, we herein demonstrated that consistent with the previous studies,^68^, ^69^, ^70^ AFL showed the ability to reduce RNV, but not to inhibit VO. However, tFNAs dramatically inhibited VO in OIR retinas, which was superior to AFL. Interestingly, we found that VO area was significantly decreased after tFNAs treatment and regenerated superficial vascular plexus could be seen.

In an effort to better understand the molecular and cellular mechanisms underlying the effects of tFNAs in the treatment of ischaemic retinopathy, we attempted to study the PI3K/AKT/mTOR signalling pathway. Western blot and cell immunofluorescence staining analysis of the expression of proteins related to the PI3K/AKT/mTOR signalling pathways in vitro confirmed that tFNAs might inhibit angiogenesis through this signalling pathway. Our previous studies showed that tFNAs could activate the PI3K/AKT/mTOR pathway to enhance the autophagy, proliferation and migration of cells and promote angiogenesis, wound healing and neuroprotective effects.^38^, ^56^, ^71^, ^72^, ^73^, ^74^

In our study, we found that the number of filopodia and tip cells were significantly increased in tFNAs‐treated cells, but not in AFL‐treated cells. As documented, filopodia protrusions have been proposed to drive endothelial tip cell functions by translating guidance cues into directional migration and mediating new contacts during anastomosis, thus the number of filopodia and tip cells could be served as a biomarker of vasculogenesis.^75^, ^76^, ^77^ Taken together, these results indicated that, during hypoxia, tFNAs mainly suppressed the PI3K/AKT/mTOR pathway, but not VEGF/VEGFR pathway, thus, inhibited endothelial cell migration, tube formation and cell proliferation in vitro. Even more interestingly, as aforementioned, tFNAs‐treated OIR mice were beneficial even more for ameliorating retinal VO and promoting the normalization of disrupted vasculature.

Based on these, we speculated that tFNAs might act as a novel and potential mTOR inhibitor. Our results showed that tFNAs reduced the extent of pathological retinal neovascular tufts significantly, which were consistent with those in previous studies on direct/indirect mTOR inhibitor (rapamycin or valproic acid, respectively).^78^, ^79^ In addition, Yagasaki et al. compared the effects of mTOR inhibitor (rapamycin) with VEGFR blocker (KRN633) on retinal vascular development, and found that inhibition of vascular development, characterized by attenuation of retinal vascularization and decreased spouts of the superficial vessels growing, was seen followed by KRN633 treatment, but not by rapamycin treatment.^80^ These results indicated that, unlike VEGFR blocker, mTOR inhibitor did not significantly disturb nor delay the physiological retinal angiogenesis. Similarly, in our current study, tFNAs were administered at P12 (the late stage of retinal vessel development). Our results indicated that, unlike AFL, normal retinal development was not delayed or compromised while RNV was inhibited after tFNAs treatment. Moreover, we hypothesized that as positive feedback, the reduction of hypoxic avascular area suppressed the VEGF, alleviated the hypoxia ischemia in retina, thereby further reducing RNV formation.

## 5 CONCLUSION

In summary, our results showed that tFNAs could safely and efficiently enter the vascular endothelial cells under hypoxia condition, could blunt proliferation, migration and tube formation of HUVECs in vitro, thus, inhibit pathological RNV. Our findings in OIR mice suggested that tFNAs is beneficial for not only inhibiting pathological neovascularization, but also suppressing retinal VO and promoting the normalization of disrupted vasculature in the ischaemic retina. It is shown that tFNAs mainly regulate the PI3K/AKT/mTOR signalling pathway to inhibit endothelial cell growth and metabolism under hypoxia. Our study highlighted the novel and potential opportunity of tFNAs for treatment of ischaemic retinal diseases, a leading cause of blindness in developed and developing countries worldwide.

## AUTHOR CONTRIBUTIONS

*Study conception and design*: Xiaodi Zhou, Xiaoyan Ding, Yunfeng Lin. *Acquisition of data*: Xiaodi Zhou, Yanting Lai, Xiaoxia Xu, Limei Sun, Limei Chen. *Analysis and interpretation of data*: Xiaodi Zhou, Yanting Lai, Xiaoxiao Xu, Qiong Wang, Jiajie Li, Rong Li, Delun Luo. Xiaodi Zhou, Yanting Lai and Xiaoxiao Xu contributed equally to this work. All authors reviewed the results and approved the final version of the manuscript.

## CONFLICT OF INTEREST STATEMENT

The authors declare no conflict of interest.

## Data Availability

The data that support the findings of this study are available from the corresponding author upon reasonable request.
